# Trajectory of smoking and early bladder cancer risk among Korean young adult men

**DOI:** 10.1007/s10552-020-01335-8

**Published:** 2020-08-18

**Authors:** Yongho Jee, Keum Ji Jung, Joung Hwan Back, Sun Mi Lee, Seung Hwan Lee

**Affiliations:** 1 Advanced Biomedical Research Institute, Ewha Womens University Seoul Hospital, Seoul, South Korea; 2 Institute for Health Promotion, Graduate School of Public Health, Yonsei University, Seoul, South Korea; 3 Health Insurance Policy Research Institute, Wonju, South Korea; 4 Department of Urology, Urological Science Institute, Yonsei University College of Medicine, Seoul, South Korea

**Keywords:** Smoking, Trajectory, Bladder cancer, Young adults

## Abstract

**Objectives:**

The aim of this study was to examine the risk of bladder cancer according to the trajectory pattern of amount of smoking among Korean young adult men.

**Methods:**

Smoking status was assessed with a standardized questionnaire in the Korean Life Course Health Study (KLCHS). Trajectory analyses were performed among young adult men using seven repeated surveys of cigarette per day (CPD) every two years from 1992 to 2005. The occurrence of bladder cancer was tracked from 2006 to 2016. The Cox proportional models were used to calculate the hazard ratio (HR) (95% confidence interval) of smoking patterns on bladder cancer.

**Results:**

The mean (standard deviation) age of the 161,069 participants was 34.0 (3.9) years, and 2,280,143 person-years (PY) were examined during the follow-up period of 14.2 (median 14.3) years. During this period, 263 new cases of bladder cancer occurred (11.5/100,000 PY). Among the six trajectory groups (low steady, lowering, rise and fall, high steady, rise and sharp fall, and very high steady), there was a higher risk of developing bladder cancer in the all the other groups compared to the low steady group. The highest risk group was the very high steady group, with HR 2.83 (95% CI 1.79–4.49). In addition, the risk of bladder cancer was 2.61 (95% CI 1.50–4.54) in the rise and sharp fall group.

**Conclusion:**

The risk of bladder cancer did not show much difference according to trajectories, except for low steady group. Thus quitting smoking should be the priority to lower the risk of bladder cancer in smokers.

**Electronic supplementary material:**

The online version of this article (10.1007/s10552-020-01335-8) contains supplementary material, which is available to authorized users.

## Introduction

While bladder cancer is a relatively rare cancer in Korea, globally the incidence is gradually increasing and has been reported to have a poor prognosis. In previous studies, several risk factors were identified for early diagnosis or prevention of this type of cancer. Smoking [1], coffee consumption [2, 3], occupational exposure [4], and dietary factors [5] constitute the most important exogenous risk factors for bladder carcinogenesis [6]. Among these, smoking is the most well-known risk factor for bladder cancer [7, 8].

A meta-analysis of the association between smoking and bladder cancer in 89 observational studies showed that the summary odds ratio of bladder cancer incidence was 3.14 times (2.53–3.75) in current smokers and 1.83 (1.52–2.14) times in former smokers [9]. Moreover, it has been reported that second-hand smoking increases the risk of bladder cancer in non-smokers [10].

Association between coffee consumption and bladder cancer remains controversial.

Case–control study conducted by Yu et al. reported the positive association between coffee consumption and bladder cancer among never smokers but not in current smokers [3]. Meta-analysis published by Dai et al. reported that to date available evidence was insufficient to support an independent association between coffee consumption and bladder cancer risk [2]. Although, there was an increased risk of bladder cancer related to higher coffee consumption among studies with fewer cases (RR high vs low = 1.38, 95% CI 1.05–1.81), smoking was poorly adjusted among these studies.

The previous studies have the following characteristics. First, they were mostly case–control studies with some exceptions [11]. Second, many studies have examined the association with bladder cancer by measuring smoking once at the baseline. However, smoking pattern has the characteristic of changing over time, especially in young adults [12]. Third, the study subjects were mostly middle-aged and elderly. However, in fact, a rapid increase in the smoking rate is observed in young people in their 20s and 30s [13, 14].

Therefore, it is meaningful to investigate the high rate of smoking in young people and the risk of developing bladder cancer in the middle-aged population. In particular, reports on the long-term health effects according to the pattern of smoking behavior examined multiple times in young individuals are rare. Moreover, the effect of repeatedly measured smoking status analyzed by group-based trajectory on bladder cancer remains uncertain. Therefore, our aim was to analyze the trajectory of smoking in young adults and analyze the bladder cancer incidence in each trajectory group.

## Methods

### Study population

This study is part of the Korean Life Course Health Study (KLCHS), which has been described previously [15]. The study population was selected from the national health examination conducted on a biennial basis for Korean civil and private school staff since 1992. From 1992 to 1999, health examination by Korea medical insurance corporation (KMIC) was provided in even years to insured persons and in odd years to dependents. A total of 4,862,438 (10.7%) members of the Korean population were covered by KMIC insurance; among them, 1,297,833 were employees and 3,364,405 were dependents. Since 2000, coverage for even-numbered and odd-year births have been provided health examination by National Health Insurance Service (NHIS).

Even-numbered and odd-year births have been provided health examination by National Health Insurance Service (NHIS). The characteristics of this study can be found in the previously reported Korean Cancer Prevention Study (KCPS) [14, 15]

For this study, 430,951 individuals (307,652 males, 123,299 females) aged 20–39 years were selected based on their ages in 1992 and 1993. Among them, the smoking rate in women was extremely low (0.2%), and they were excluded. Of the 307,652 men, 298,440 were included in the study at baseline, with the exception of 9,212, who were missing in the 1992–1993 survey. The following two-year screenings included 274,978 in 1994–1995; 252,209 in 1996–1997; 231,829 in 1998–1999; 197,006 in 2000–2001; 177,467 in 2002–2003; and 161,098 persons in 2004–2005. Of the 161,098 participants, 29 were excluded for having already developed cancer, so the final sample was 161,069 men. The study proposal was approved by the Institutional Review Board of Human Research, Yonsei University (4-2001-0029).

### Smoking behavior and other covariate data

All participants were given the opportunity to undergo a medical examination every two years since 1992. The smoking history data were obtained by a self-administered questionnaire. Cigarette smoking history was coded from 1 to 5. The smoking variables in this study were divided into five groups as follows: 1 = non-smokers, 2 = ex-smokers, 3 = 1–9 cigarettes per day, 4 = 10–19 cigarettes per day, and 5 = more than 20 cigarettes per day. This analysis was performed assuming an interval variable. These surveys on smoking amount were conducted every two years from 1992 to 2005. Height, weight, alcohol intake, and exercise habit of all participants were recorded to describe the characteristics of the participants. Height, weight and alcohol intake were recorded by professionally trained investigator. Information on exercise was obtained by asking “Do you exercise regularly?” (Yes/No). If participants answered “Yes,” they were asked to indicate the frequency of exercise per week on continuous scale. In this study, we merely used dichotomous variable.

Bladder cancer follow-up

The occurrence of bladder cancer was tracked from 2006 to 2016. Bladder cancer (ICD-10 code C67) was confirmed through the National Cancer Registry of the Korean National Cancer Center.

### Statistical analysis

Our study analyzed the smoking pattern in 161,069 young people aged 20–39 years through the trajectory analysis of smoking, measured seven times over 14 years from 1992 to 2005. Trajectory analyses were performed using seven repeated surveys of cigarette per day, using the PROC TRAJ command in SAS [17, 18]. PROC TRAJ is a SAS Trajectory for group-based modeling of longitudinal data (SAS Institute, Inc., Cary, NC, USA)

**Fig. 1:**
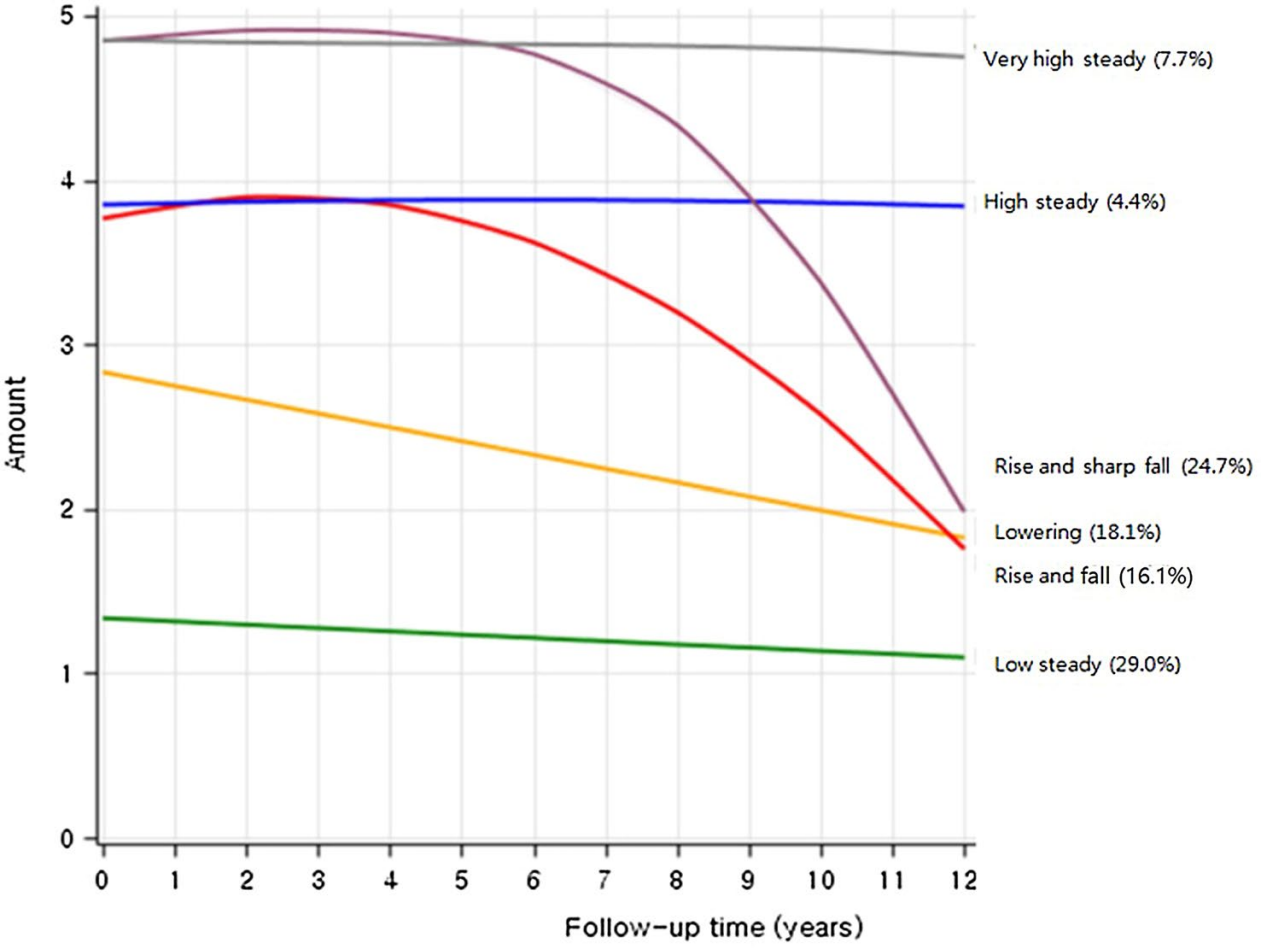
Trajectory group of smoking amounts in Korean young adult men

Trajectory analysis uses semi parametric group-based modeling strategies to identify potential patterns of end-to-end data. Each model represents an individual with a similar trajectory [18]. Using Bayesian Information Criterion (BIC) to assess the best model fit, number of trajectory groups were limited to less than six [19, 20]. We grouped the name according to the characteristics of the five trajectory groups shown in Fig. 1 as follows: Group 1 (29.0%), ‘low steady’ refers to the participants who were maintained low smoking amount; Group 2 (18.1%), ‘lowering’ refers to participants who steadily reduced their smoking amount; Group 3 (4.4%), ‘high steady’ refers to participants who showed steadily rising smoking amount; Group 4 (24.7%), ‘rise and sharp fall’ refers to participants who initially had high smoking amount and showed sharped decreased after the 4-year follow-up.

Group 5 (16.1%), ‘rise and fall’ refers to participants who initially increased their smoking amount and decreased later; Group 6 (7.7%), ‘very high steady’ refers to those who kept on maintaining high smoking amount.

The association between smoking history and bladder cancer was analyzed in three models. We compared Model 1 and Model 2 to clarify the risk of bladder cancer according to the smoking status in the baseline period (Model 1) and the smoking status in the intermediate period (Model 2). Model 1 analyzed bladder cancer incidence according to the amount of smoking in 1992–1993. Model 2 analyzed bladder cancer incidence according to the amount of smoking in 2004–2005. Model 3 analyzed bladder cancer incidence according to the group finally adopted in trajectory analysis during 1992–2005. Various models, including smoking measured once and smoking trajectory, were evaluated as Akaike information criteria (AIC) values. Time dependent analysis was performed in consideration of lag time in Model 4. The relative risk of smoking was analyzed by the Cox proportional hazard model including age, body mass index (BMI), and alcohol consumption. Statistical analysis was performed using SAS 9.4.

## Results

Table 1 compares the general characteristics of the 161,069 participants included in the final analysis of this study with the 146,583 participants not included. The mean age, BMI, and alcohol consumption of the two groups were statistically different. Also, the overall distribution of smoking amount showed similar trends but were statistically different; exercise, in contrast, showed no difference. Among the 161,069 participants, the average age (standard deviation) was 34.0 (3.9) years. Participants were observed for 2,280,143 person-years during the average follow-up period of 14.2 (median 14.3) years. During this period, 263 new cases of bladder cancer (incidence 11.5/100,000 PY) occurred. By age group, there were 13 cases in 20–29 year olds (7.7/100,000 PY), 71 cases in 30–39 year olds (7.3/100,000 PY), and 179 cases in 40–49 year olds (16.3/100,000 PY). During the follow-up period, total deaths were 3,461, of which 23 were due to bladder cancer.

**Table 1:** General characteristics of the groups included and not included in the study at baseline (1992–1993)

	Included	Excluded	*p* value
*N*	161,069	146,583	
Mean age, years (SE)	34.0 (3.9)	33.5 (4.2)	< 0.0001
Body mass index, kg/m^2^ (SE)	23.0 (2.5)	23.1 (2.5)	< 0.0001
Alcohol intake, g per day (SE)	17.2 (20.7)	19.0 (23.9)	< 0.0001
Smoking amount, cigs. per day			
Non-smoker	20.2%	20.9%	< 0.0001
Ex-smoker	16.9%	15.6%	
1–9	16.9%	16.2%	
10–19	28.2%	27.1%	
20 +	18.0%	20.2%	
Exercise			
No 1	79.9%	79.9%	0.6880
Yes 2	20.1%	20.1%	

Table 2 shows the general characteristics of the six trajectory groups (low steady, lowering, rise and fall, high steady, rise and sharp fall, and very high steady) from the smoking amount record measured every two years from 1992 to 2005 (bottom and right in Fig. 1). In the ‘low steady’ group, which had a lower smoking rate, 66.9% were non-smokers in 1992–1993 and 30.1% were ex-smokers. In 2004–2005, most of them were reported as non-smokers, with a non-smoking rate was 91.8%. In contrast, the ‘very high steady’ group had a very low non-smoking rate in 1992–1993 (0.3%), and 82.7% of the groups smoked more than 20 cigarettes per day. In 2004–2005, 71.5% of the respondents smoked more than 20 cigarettes per day.

**Table 2:** General characteristics by trajectory group based on the smoking amount recorded every two years from 1992 to 2005

	Low steady	Lowering	Rise and fall	High steady	Rise and sharp fall	Very high steady
*N* (%)	46,756 (29.0)	29,101 (18.1)	25,969 (16.1)	39,741 (24.7)	7,100 (4.4)	12,402 (7.7)
Age, mean (SD), years	34.1 (4.0)	34.3 (3.9)	34.0 (3.9)	33.7 (4.0)	34.3 (3.9)	33.9 (4.0)
Weight, kg (SD)	66.2 (8.0)	66.8 (8.0)	66.9 (8.2)	66.4 (8.2)	66.9 (8.5)	68.2 (8.5)
Height, cm (SD)	169.8 (4.9)	170.4 (4.9)	170.4 (4.9)	170.3 (4.9)	170.6 (4.8)	170.5 (4.8)
Body mass index, kg/m^2^ (SD)	22.9 (2.4)	23.0 (2.4)	23.0 (2.4)	22.9 (2.4)	23.5 (2.5)	23.4 (2.5)
Alcohol amount, cig/day (SD)	11.0 (15.9)	16.8 (19.3)	19.2 (21.0)	19.6 (20.9)	24.4 (26.1)	25.5 (27.2)
Smoking in 1992–1993, *N* (%)						
Non	31,297 (66.9)	536 (1.8)	190 (0.7)	508 (1.3)	4 (0.1)	29 (0.3)
Ex	14,074 (30.1)	9,817 (33.7)	1,305 (5.0)	1,590 (4.0)	35 (0.5)	70 (0.6)
1–9	1,200 (2.6)	11,410 (39.2)	6,162 (23.8)	8,109 (20.4)	70 (1.0)	216 (1.7)
10–19	147 (0.3)	5,743 (19.7)	13,846 (53.3)	22,857 (57.5)	1,039 (14.6)	1,829 (14.8)
20 +	38 (0.1)	1,595 (5.5)	4,466 (17.2)	6,677 (16.8)	5,952 (83.8)	10,258 (82.7)
Smoking in 1998–1999, *N* (%)						
Non	41,819 (89.4)	5,188 (17.9)	533 (2.1)	897 (2.3)	25 (0.3)	59 (0.5)
Ex	4,477 (9.6)	13,044 (44.8)	2,499 (9.6)	1,566 (3.9)	52 (0.7)	86 (0.7)
1–9	367 (0.8)	8,385 (28.8)	2,740 (10.6)	2,482 (6.3)	24 (0.3)	30 (0.2)
10–19	74 (0.2)	2,357 (8.1)	17,451 (67.2)	28,572 (71.9)	1,235 (17.4)	1,364 (11.0)
20 +	19 (0.04)	127 (0.4)	2,746 (10.6)	6,224 (15.7)	5,766 (81.2)	10,866 (87.6)
Smoking in 2004–2005, *N* (%)						
Non	42,927 (91.8)	9,399 (32.3)	9,592 (36.9)	114 (0.3)	2,513 (35.4)	0 (0.0)
Ex	3,093 (6.6)	13,826 (47.5)	13,458 (51.8)	1,218 (3.1)	3,434 (48.4)	0 (0.0)
1–9	602 (1.3)	4,617 (15.9)	2,559 (9.9)	4,336 (10.9)	336 (4.7)	129 (1.0)
10–19	112 (0.2)	1,177 (4.0)	365 (1.4)	29,287 (73.7)	799 (11.3)	3,406 (27.5)
20 +	22 (0.1)	82 (0.3)	0 (0.0)	4,786 (12.0)	18 (0.3)	8,867 (71.5)

There were two groups with high smoking rates and then sudden declines. First, ‘the rise and fall’ group had a high smoking rate in 1992–1993, with 17.2% of those in 20 or more and 0% in 20 or more in 2004–2005. The ‘rise and sharp fall’ group, in contrast, was already high at 83.8% in 1992–1993 and dropped sharply to 0.3% in 2004–2005.

Table 3 shows the association between smoking and bladder cancer in the four models. Model 1 shows the association between smoking status in 1992–1993 and bladder cancer. We made an examination using different smoking status period in Model 2 analyzing the association between smoking status in 2004–2005 and bladder cancer.

**Table 3:** Smoking and bladder cancer risk in three models (Cox proportional hazard model)

	Classic approach		Trajectory approach	Time dependent analysis
	Model 1	Model 2	Model 3	Model 4
Smoking in 1992–1993				
Non	1.0			
Ex	1.08 (0.65 to 1.79)			
1–9	2.36 (1.53 to 3.64)			
10–19	2.17 (1.45 to 3.25)			
20 +	2.54 (1.69 to 3.84)			
Smoking in 2004–2005				
Non		1.0		
Ex		1.40 (1.02 to 1.93)		
1–9		1.28 (0.80 to 2.04)		
10–19		1.67 (1.39 to 2.51)		
20 +		1.43 (0.91 to 2.24)		
Trajectory in 1992–2005				
Low steady			1.0	
Lowering			1.59 (1.06 to 2.38)	
Rise and fall			1.40 (1.34 to 2.97)	
High steady			2.21 (1.55 to 3.15)	
Rise and sharp fall			2.52 (1.50 to 4.23)	
Very high steady			2.76 (1.77 to 4.31)	
Time varying smoking				
Non				1.0
Current smoking				2.50 (1.73 to 3.61)
Bladder cancer/N	294/161,064	294/161,064	294/161,064	281/161,051
Degree of freedom	7	7	8	4
Akaike’s Information Criteria	6564.443	6485.267	6472.382	6447.946

*Adjusted for age, body mass index, and alcohol amount; Participants (*n* = 85) with missing records on smoking amount were excluded

AIC Difference between model 3 and model 4 was 24.436 (*p* value < 0.001)

Model 3 compared the association of bladder cancer with the trajectory group based on the smoking amount at 7 time-points in 1992–2005. Since the smoking status was measured in 1992–1993, the hazard ratio (95% confidence interval [CI]) of bladder cancer in the ex-smokers compared to non-smokers was 1.08 (0.65 to 1.79), which was not significant. However, in 1992–1993, when the amount of smoking was 1–9 cig per day, the hazard ratio (95% CI) of bladder cancer was 2.36 (1.53 to 3.64), 2.17 (1.53 to 3.64) for 10–19 cig per day, and 2.54 (1.69 to 3.84) for more than 20 cigs per day, which were all significant.

Model 2 analyzes the association between the smoking amount in 2004–2005 and the risk of bladder cancer during 2005–2016. Compared to non-smokers, ex-smokers were 1.40 times more likely to develop bladder cancer. In addition, current smokers with 10–19 cig per day were 1.67 times more likely to develop bladder cancer.

Model 3 (AIC = 6472.382), which analyzed the association between the trajectory group of smoking amount and bladder cancer incidence, showed better performance than did model 2 (AIC = 6485.267). Among the six trajectory groups, there was a higher risk of developing bladder cancer in the other groups compared to the ‘low steady’ group. The highest risk group was ‘very high steady’ group with a hazard ratio = 2.76 (1.77 to 4.31). In addition, the risk of bladder cancer was 2.52 (95% CI 1.50 to 4.23) in the rise and sharp fall group.

Model 4 shows the time varying effect of smoking on bladder cancer, the performance was better than the trajectory model (AIC = 6472.382). Time varying current smoking raised the risk of bladder cancer by 2.50 (95% CI 1.73 to 3.61) times higher than non-smoking.

## Discussion

Through trajectory analysis, our study analyzed the patterns of the smoking amount measured 7 times during 14 years for people aged 20–39 years, in whom high smoking prevalence is observed during their lifetime. We also analyzed the relationship between trajectory group and bladder cancer. As a result, the groups with the highest smoking amounts (high steady and very high steady) showed more than twice the risk of bladder cancer. Further, the rise and fall and the rise and sharp fall groups still showed more than double the risk.

Our results were similar to the results of the meta-analysis reported in 2018 [1, 9]. In the meta-analysis, current smokers showed 3.14 times higher risk than non-smokers, while the risk of very high steady group was 2.83 higher in our study. The conclusion of the meta-analysis was that active smokers are at an increased risk of bladder cancer.

“However, due to heterogeneity between the population used in our study and population of studies involved in the meta-analysis, we should conduct cautious interpretation to compare the results in two studies. While the studies involved in the meta-analysis analyzed the risk of bladder cancer in age 70s, participants used in our study was relatively young, therefore we were allowed to analyze the risk of bladder cancer when the participants became middle-aged.” Dose–response meta-analyses showed a bladder cancer risk plateau for smoking intensity; this indicates that even after long-term smoking cessation, the risk of bladder cancer remains high [1, 9] In the meta-analysis, the risk of bladder cancer of the group that quit smoking was 1.83 times higher risk compared with never smokers. In our study, the risk of the group that continued to show a high smoking rate but later abruptly quit was 1.94 to 2.61 times higher. However, during the smoking history period, the lowering group, in which individuals continued to reduce the smoking amount from the beginning, did not show a significantly increased bladder cancer risk (hazard ratio, 1.50, 95% CI 0.98 to 2.30).

The trajectory analysis in this study is also different from other reports, in which the smoking cessation group was subdivided into three heterogeneous groups to analyze the effects of bladder cancer. This study used both a classic epidemiologic study approach and a group-based trajectory approach for smoking and bladder cancer. In other words, the analysis between 2006 and 2016 according to the smoking amount in 1992–1993 (model 1 in Table 3) and the analysis between 2006 and 2016 according to smoking history in 2004–2005 (model 2 in Table 3) were the analyses of the classic approach. Model 1 and Model 2 could not directly compare the AIC values for performance because the number of subjects included in the analysis was different. However, comparing model 2 with the trajectory model 3, the performance of model 3 was significantly better.

Smoking and other lifestyle factors are the most important exogenous risk factors for bladder cancer. However, individuals with seemingly equal exposure to environmental risk factors develop bladder cancer in a heterogeneous pattern. This is probably attributed to the fact that genetic background varies in human populations, pointing to the role of genetic susceptibility in human cancer [6]. Smoking accounts for approximately 52% of the bladder cancer incidence among postmenopausal women, but the underlying mechanism is poorly understood. There is a recent study on whether DNA methylation levels in the blood predict the development of bladder cancer [6, 16]. They suggested that differential methylation of cg05575921 and cg19859270 mediate the effects of smoking on bladder cancer, potentially revealing downstream effects of smoking relevant for carcinogenesis.

In the present study, since the trajectory analysis included only those subjects with all seven results, whose smoking history was examined from 1992 to 2004, it was important to compare the general characteristics of these differences (Table 1). In both groups, there were statistically significant differences in age, BMI, alcohol consumption, and smoking, but the overall mean and distributions were similar. This can be observed as the statistical significance due to the large sample. Thus, although it may be interpreted that there was a slight selection bias in the entire population, it did not seem to have a significant effect on the study results.

The strength of this study was that trajectory groups were formed using seven repeatedly measured smoking data over a 12-year period. However, while such a long follow-up time was an advantage [17, 18], there was a limitation in that those who did not participate in every survey had to be excluded because the amount of smoking was missing. Another limitation was that smoking data were measured as interval variables rather than continuous variables. Despite these limitations, the overall result is considered to be justified in the high risk of bladder cancer in the group that continued to maintain high levels of smoking amounts. Another limitation was that the study did not include variables for fluid intake and socio-economical status in multivariate analysis.

This study analyzed only males because smoking rates among females were very low.

Recently, the risk of smoking has been observed to be high in women with bladder cancer, and the interaction between smoking and sex has been reported [21]. Therefore, a sex interaction study on bladder cancer would be an important topic for future studies.

In conclusion, the smoking status measured at the baseline may change over time. With this in mind, analyzing trajectory provides information on important implications for understanding health-related concerns throughout life. Those who maintained high steady smoking had a higher risk of developing bladder cancer. Quitting smoking, or reducing the smoking amount, can be effective in reducing the risk of bladder cancer.

## Electronic supplementary material

Below is the link to the electronic supplementary material.

Supplementary file1 (DOCX 185 kb)
