# Supplementary material for: Trajectory of smoking and early bladder cancer risk among Korean young adult men
Source: Cancer Causes Control. 2020 Aug 18;31(10):943–9. doi: 10.1007/s10552-020-01335-8 (PMC7458885; doi:10.1007/s10552-020-01335-8)
Supplement: Supplementary file 1 — Supplementary file1 (DOCX 185 kb) [file 10552_2020_1335_MOESM1_ESM.docx]

| Smoking status |  | | Hazard Ratio |
| --- | --- | --- | --- |
| Non-smoker in 2004 | | | 1.0 |
| Ex-smoker (recent quitting) in 2004 | | | 1.02 (0.72 to 1.45) |
| 1-9 cigarettes per day in 2004 | |  | 0.89 (0.54 to 1.46) |
| 10-19 cigarettes per day in 2004 | | | 1.22 (0.87 to 1.73) |
| 20+ cigarettes per day in 2004 | | | 0.88 (0.54 to 1.44) |
| Non-smoker in 1992 | | | 1.0 |
| Ex-smoker in 1992 (long-term quitting) | | | 1.07 (0.63-1.80) |
| 1-9 cigarettes per day in 1992 | | | 2.29 (1.42-3.70) |
| 10-19 in 1992 | | | 2.02 (1.27-3.20) |
| 20+ in 1992 | | | 2.45 (1.52-3.94) |

Supplementary table 1. Comparison between the effects of recent quitting versus long-term quitter

| Trajectory shapes: 02, BIC=51719.6 | Trajectory shapes: 012, BIC=49213.9 | Trajectory shapes: 0122, BIC=47885.2 |
| --- | --- | --- |
| 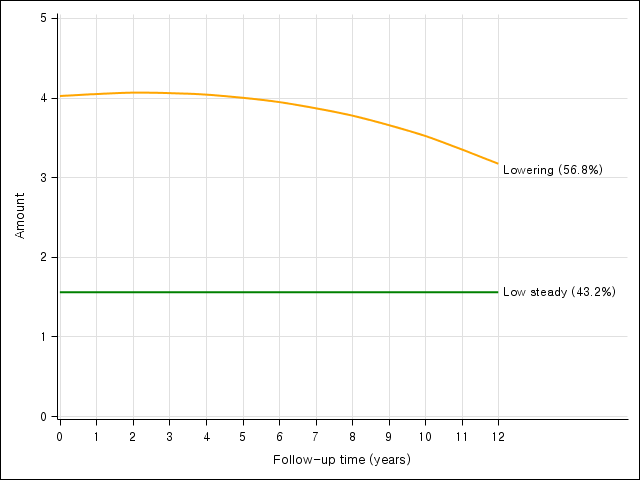 | 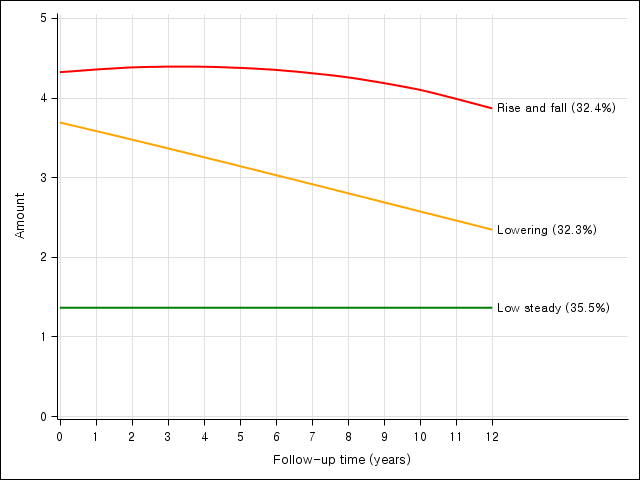 | 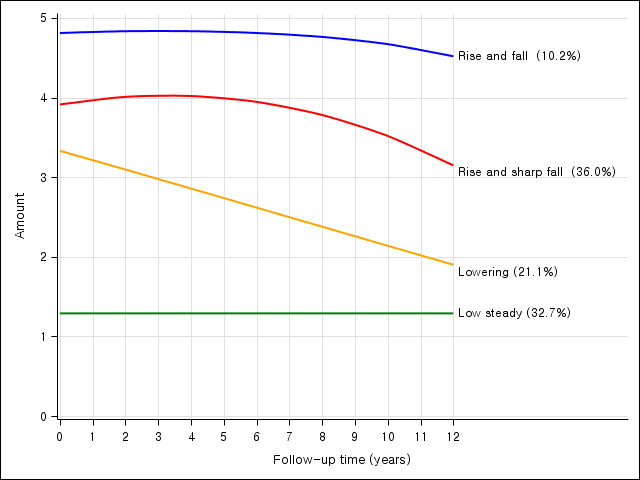 |
| Trajectory shapes: 0123, BIC=47888.6 | Trajectory shapes: 01222, BIC=46946.8 | Trajectory shapes: 112223, BIC=46429.5 |
| 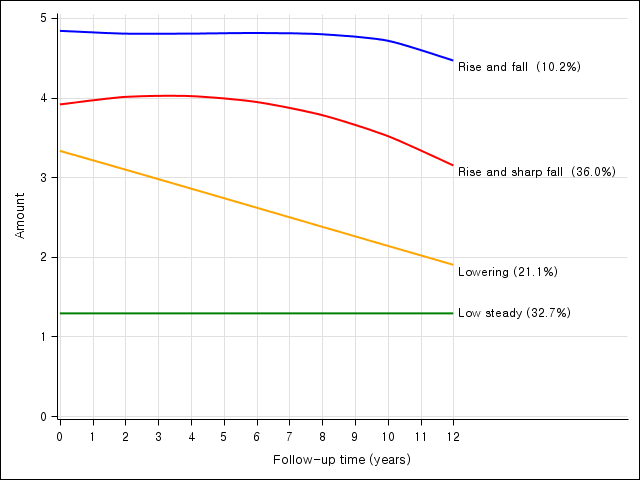 | 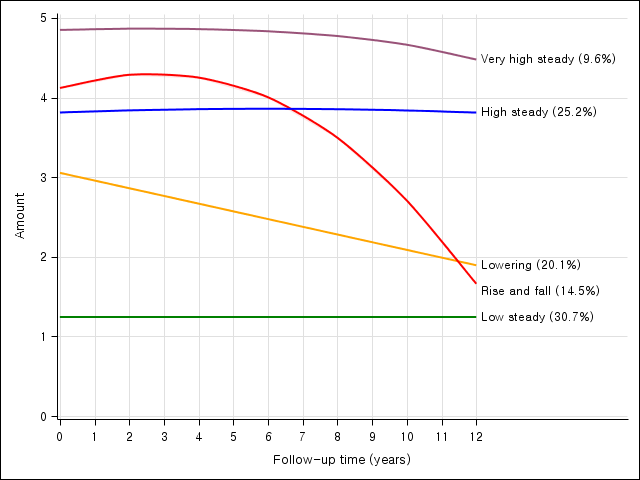 | 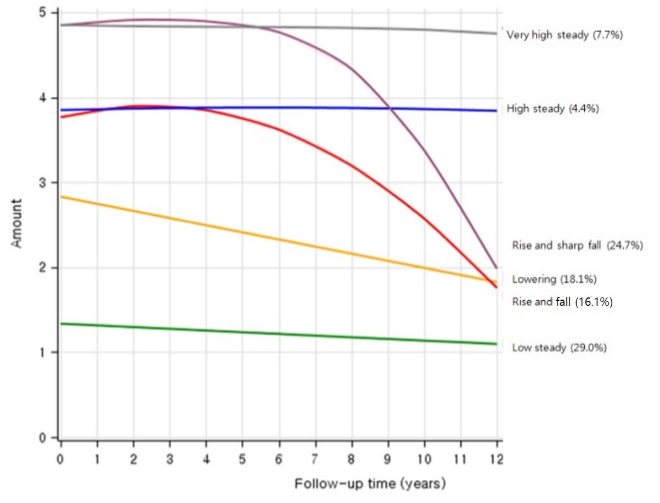 |

Supplementary Figure 1. Trajectory group of smoking amount for 6 models

Group 1

Low steady

Bladder Cancer

incidence

Baseline Smoking

- Non-smokers

- Former-smokers

- Current smokers

: 1-9 cig/day

: 10-19 cig/day

: ≥20 cig/day

Group 5

Rise and fall

Group 4

Rise and sharp fall

Group 3

High steady

Group 2

Lowering

1992-1994

1992-2004

2006-2016

Group 6

Very high steady

Supplementary Figure 2. Trajectory model of smoking amount during 12 years (1992-2005)
